# An Adaptation-Induced Repulsion Illusion in Tactile Spatial Perception

**DOI:** 10.3389/fnhum.2017.00331

**Published:** 2017-06-28

**Authors:** Lux Li, Arielle Chan, Shah M. Iqbal, Daniel Goldreich

**Affiliations:** ^1^Department of Psychology, Neuroscience and Behaviour, McMaster UniversityHamilton, ON, Canada; ^2^McMaster Integrative Neuroscience Discovery and Study, McMaster UniversityHamilton, ON, Canada

**Keywords:** somatosensory, psychophysics, sensory adaptation, perceptual inference, tactile illusion, two-point perception, human, aftereffect

## Abstract

Following focal sensory adaptation, the perceived separation between visual stimuli that straddle the adapted region is often exaggerated. For instance, in the tilt aftereffect illusion, adaptation to tilted lines causes subsequently viewed lines with nearby orientations to be perceptually repelled from the adapted orientation. Repulsion illusions in the nonvisual senses have been less studied. Here, we investigated whether adaptation induces a repulsion illusion in tactile spatial perception. In a two-interval forced-choice task, participants compared the perceived separation between two point-stimuli applied on the forearms successively. Separation distance was constant on one arm (the reference) and varied on the other arm (the comparison). In Experiment 1, we took three consecutive baseline measurements, verifying that in the absence of manipulation, participants’ distance perception was unbiased across arms and stable across experimental blocks. In Experiment 2, we vibrated a region of skin on the reference arm, verifying that this focally reduced tactile sensitivity, as indicated by elevated monofilament detection thresholds. In Experiment 3, we applied vibration between the two reference points in our distance perception protocol and discovered that this caused an illusory increase in the separation between the points. We conclude that focal adaptation induces a repulsion aftereffect illusion in tactile spatial perception. The illusion provides clues as to how the tactile system represents spatial information. The analogous repulsion aftereffects caused by adaptation in different stimulus domains and sensory systems may point to fundamentally similar strategies for dynamic sensory coding.

## Introduction

Prolonged exposure to stimulation causes a reduction in neuronal firing rate. For reasons that have yet to be elucidated, this phenomenon, adaptation, is ubiquitous in neural sensory systems (Wark et al., 2007; Sato and Aihara, 2011). Adaptation may have several beneficial consequences: it may support perceptual constancy, increase the salience of novel stimuli, improve discrimination and improve coding efficiency (for review see Webster, 2012).

A seemingly non-beneficial consequence of focal adaptation is that it produces illusions. For instance, following focal adaptation, the perceived separation between stimuli that straddle the adapted region is often exaggerated. A well-known example of this is the visual tilt after effect illusion: adaptation to tilted lines causes subsequently viewed lines with nearby orientations to appear tilted away, i.e., repelled, from the adapted orientation (Gibson and Radner, 1937; Magnussen and Johnsen, 1986; Dragoi et al., 2000, 2001; He and MacLeod, 2001).

In vision, adaptation-induced repulsion illusions have been reported to affect perception of a wide variety of stimulus features, including luminance, contrast, spatial frequency, temporal frequency, color, contour, shape, size, orientation, motion direction, contingent visual properties (e.g., color and orientation, as in the McCollough effect) and high-level features such as the gender, ethnicity and emotion of faces (for reviews, see Clifford et al., 2007; Kohn, 2007; Webster, 2012). Adaptation-induced repulsive aftereffects have also been reported in auditory perception and audio-visual perception, including aftereffects in sound localization (Thurlow and Jack, 1973; Kashino and Nishida, 1998; Carlile et al., 2001), duration (Walker et al., 1981; Heron et al., 2012), loudness (Kitagawa and Ichihara, 2002), and high-level auditory perception such as action sounds (Barraclough et al., 2017).

The present study concerns a particular type of adaptation-induced repulsion illusion, *spatial* repulsion, in which the positions of stimuli are perceptually repelled away from an adapted area. Spatial repulsion illusions have been well documented in vision (Clifford et al., 2007; Kohn, 2007; Schwartz et al., 2007) and to a lesser extent in audition (Kashino and Nishida, 1998; Carlile et al., 2001) but have rarely been reported in touch. An early tactile study reported that prolonged static pressure on the forearm altered the perceived separation between parallel bars placed on adjacent skin areas in a direction consistent with perceptual repulsion (Day and Singer, 1964). A follow-up study suggested, however, that the observed effects may not have been aftereffects but rather perceptual recalibrations induced by the particular sets of comparison stimuli to which the participants were exposed (Gilbert, 1967). Here, we revisited the question of whether adaptation-induced spatial repulsion occurs in touch. Specifically, we investigated whether focal vibratory adaptation on the forearm induces a spatial repulsion illusion affecting the perceived distance between two points of contact straddling the adapted region. We hypothesized that adaptation of the mechanoreceptors in the intervening skin would decrease the overlap between the neuronal population responses elicited by the two points. Consequently, the brain would infer a greater distance between the points: a repulsion illusion.

## Materials and Methods

### Participants

Sixty-nine participants were recruited from the McMaster University community. By self-report, all participants were free of conditions that are known to impair tactile sensitivity (e.g., calluses, scars, or injuries on tested skin areas, carpel tunnel syndrome, diabetes) or perceptual processing (e.g., neurological disorders, attention deficit disorders, dyslexia). All participants had normal or corrected-to-normal vision. Of the 69 recruits, 60 passed the perceptual qualification criteria (see below). Of the 60 qualified participants, 20 took part in Experiment 1 (13 women, 7 men; 17 right-handed, 2 left-handed, 1 ambidextrous; aged 18.7–30.5 years, median age 20.7 years), 20 in Experiment 2A (13 women, 7 men; 19 right-handed, 1 left-handed; aged 18.5–22.6 years, median age 19.9 years), and 20 in Experiments 2B and 3 (12 women, 8 men; all right-handed; aged 19.1–28.8 years, median age 20.8 years). Handedness was assessed by a modified Edinburgh Handedness Inventory (Oldfield, 1971). Participants provided signed informed consent and received monetary compensation and/or course credits for their participation. This study was carried out in accordance with the recommendations of the McMaster Research Ethics Board. All subjects gave written informed consent in accordance with the Declaration of Helsinki. The protocol was approved by the McMaster Research Ethics Board.

## Experiment 1

Experiment 1 assessed whether the baseline perception of two-point distance was stable across experimental blocks and unbiased across arms. We tested participants on a two-interval forced-choice (2IFC) two-point distance comparison task to measure their baseline two-point distance perception.

### Preparation and Skin Sites Tested

**Figure 1:**
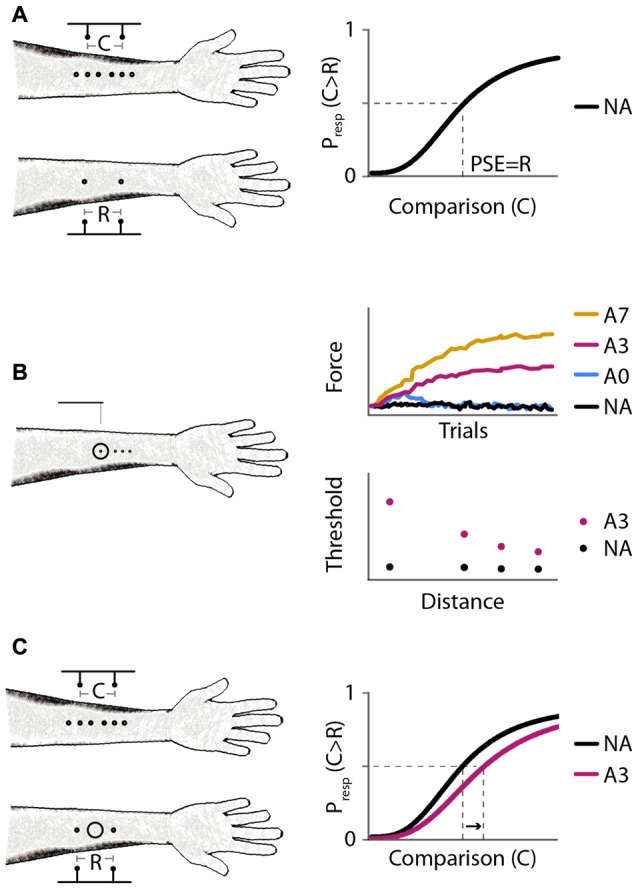
Experimental protocols and expected results. **(A)** Experiment 1: baseline distance comparison. Left: participants compared the perceived distance between two-point stimuli applied on the forearms successively. On the right arm, the points were separated by a fixed reference distance (*R* = 30 mm); on the left arm, the points were separated by a variable comparison distance (*C* = 6, 12, 18, 24, 30, 36, 42, 48, or 54 mm). Right: expected psychometric function. Horizontal axis: comparison distance, C. Vertical axis: proportion of trials in which the participant responds that C is greater than R, P_resp_(C > R). The point of subjective equality (PSE; vertical dashed line) is the value of C for which P_resp_(C > R) = 0.5 (horizontal dashed line); the expected PSE is equal to R. **(B)** Experiment 2: effect of adaptation on tactile sensitivity. Left: participants reported in which of two intervals they felt a monofilament stimulus on the right forearm. Circle: site of vibratory stimulus. Experiment 2A measured reduction in tactile sensitivity at the center of the vibration site under different adaptation protocols. Experiment 2B measured reduction in tactile sensitivity as a function of distance from the center of vibration. Right: expected results from Experiments 2A (top) and 2B (bottom). Monofilaments applied in a 2-down 1-up staircase procedure. Black, no adaptation (NA); blue, 40 s adaptation with no top-ups (A0); magenta, 40 s adaptation with 3 s top-ups (A3); yellow, 40 s adaptation with 7 s top-ups (A7). **(C**) Experiment 3: distance comparison, as in Experiment 1, but with and without adaptation. Left: circle: skin site that received vibratory adaptation. Right: expected psychometric functions. Horizontal and vertical axes as in **(A)**. Black, NA; magenta, 40 s adaptation with 3 s top-ups (A3). A rightward shift upon adaptation (arrow) indicates increased perceived distance between points straddling the adapted skin site.

The participant sat in front of a table with the experimental apparatus concealed by an opaque black curtain. The participant’s forearms, inserted under the curtain, rested comfortably on a padded surface, with the wrists (palm side up) resting stably on concave foam supports. To assist the experimenters in positioning the stimuli, the participant’s forearms were demarcated with a fine-tipped pen. A pair of small dots 30 mm apart was drawn on each volar forearm to guide the application of the two-point test stimuli. On each arm, the dots were symmetrical about the midpoint between the wrist and the elbow, aligned with the proximal-distal axis of the forearm, and slightly laterally offset from midline (Figure 1A, left). The slightly lateral-to-midline skin surface was parallel to the ground when participants rested their forearms in a supine position as they naturally tended to rotate the forearms slightly inward when relaxed; the choice of this skin surface thereby facilitated the application of the test stimuli perpendicularly to participants’ forearms.

### Psychophysical Procedure

A two-point stimulus was applied onto the participant’s volar forearm with the two points simultaneously indenting the skin. Approximately 1 s later, another two-point stimulus was applied to the other volar forearm. The participant compared the distance between the first pair of points with the distance between the second pair of points, and reported which distance felt greater (Figure 1A). The participants verbalized their answers by saying “first” or “second”, and the experimenter recorded the answers into a computer by pressing one of two response keys. The two-point distance was fixed at 30 mm on the right forearm (the reference) and variable from 6 mm to 54 mm in increments of 6 mm on the left forearm (the comparison; nine comparison distances in total). The application order of the reference and comparison points was counter balanced across participants: half of the participants received the reference points first and comparison second in all trials, and the other half of the participants received the comparison points first and reference second in all trials.

Each participant completed a practice block followed by three identical testing blocks. The practice block consisted of 16 trials with auditory feedback to indicate whether the response was correct (two trials were presented for each of the eight comparison distances not equal to the reference distance of 30 mm). Each testing block consisted of 90 trials without feedback, 10 trials at each of the nine comparison distances, randomly sampled without replacement. A custom computer program (LabVIEW 2011 for Macintosh, National Instruments) instructed the experimenter as to which comparison distance to apply. The participant took a 5 min break after the practice and a 20 min break between testing blocks. During each testing block, the participant took a 1-min break upon completing each quarter of the 90 trials (i.e., after completing trials 22, 45, 67).

### Force-Controlled Two-Point Stimuli

**Figure 2:**
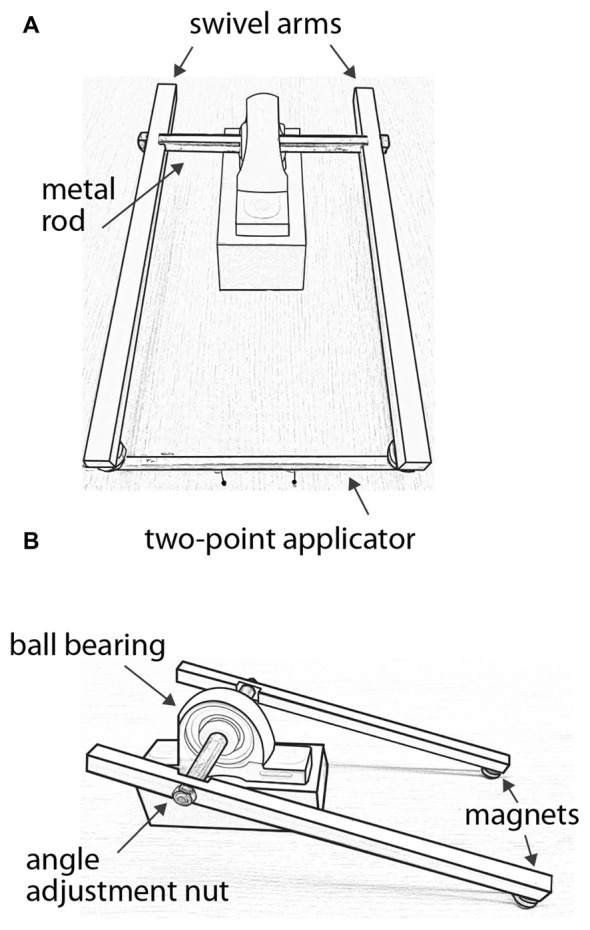
Force-controlled two-point stimulus apparatus. **(A)** Front view with a two-point applicator attached to magnets at the ends of the swivel arms. In this illustration, the applicator’s pinheads are separated by 42 mm. **(B)** Side view without applicator, illustrating the angle adjustment nut on one of the swivel arms.

A custom-made lever system (Figure 2) was used to apply two-point stimuli in alignment with the proximal-distal axis of the forearm, and with force control. Each two-point applicator was made of two plastic pins attached to one face of the shaft of a wood pencil of hexagonal cross-section. The uniform size and weight of the pencils facilitated force control of the test stimuli, and the hexagonal cross-section helped align the two pins. The heights by which the pins protruded from the pencils were carefully adjusted such that they were equal for a given two-point applicator and across all applicators. The stimulus surfaces were spherical pinheads of diameter 1.5 mm. Separation distances between the centers of the pinheads were 6, 12, 18, 24, 30, 36, 42, 48 and 54 mm.

The lever system consisted of two acetal plastic arms attached via a metal rod that passed through a ball bearing. The metal rod rotated with little friction, allowing the arms to swivel smoothly. A magnet was attached to the end of each arm, and two magnets were attached to each applicator. The applicator could be easily attached to and removed from the swivel arms via the magnets, which allowed the experimenter to quickly change the applicator from trial to trial. To apply a test stimulus, the experimenter first attached the applicator to the swivel arms. Supporting the swivel arms with both hands from below, the experimenter gently lowered the swivel arms such that the two pinheads contacted the forearm simultaneously and perpendicular to the skin surface. The pinheads contacted the skin with a total force determined by the combined weight of the swivel arms, which measured 80–82 g when the pinheads were applied with this method to a scale. The pinheads were in contact with the skin for ~0.5 s before the experimenter raised the swivel arms to end the stimulus.

Two identical lever apparatuses were used to apply the test stimuli, one for each forearm. Two experimenters were needed to conduct the experiment, each operating one lever apparatus. The order of the forearms receiving the test stimuli in each trial (either reference first or comparison first) was consistent for a given participant but counterbalanced across participants. Regardless of the order, in each trial, the stimuli were applied to the forearms sequentially. As one experimenter completed the first stimulus and raised the swivel arms away from the skin, the second experimenter initiated the stimulus to the other forearm. The inter-stimulus interval was ~1 s. The two experimenters were trained to keep the application pace consistent between stimuli and across trials.

The precise angles of the swivel arms were individually adjustable in order to match the slight change in thickness (and therefore height above the table) of the forearm along the proximal-distal axis. The experimenters adjusted the angles of the two swivel arms within each apparatus in order to ensure that the two pinheads contacted the skin simultaneously and with equal force, as reported by the participant.

### Qualification Criteria

To ensure that participants’ baseline two-point distance perception was sufficiently accurate to perform the two-point distance comparison task, we compared participants’ baseline performance in the first testing block to two qualification criteria: the proportion of “comparison is longer” responses at the longest comparison distance (54 mm) should be ≥0.7, and at the shortest comparison distance (6 mm) should be ≤0.3. If a participant failed to meet either criterion, then we considered their baseline performance as unreliable. In this case, the participant did not proceed with the experiment, and their data were excluded from analysis.

### Psychometric Function Parameterization and Estimation of Point of Subjective Equality (PSE)

For each of the three testing blocks for each participant, we fit to the data a sigmoidal cumulative normal function, which describes the proportion of trials at which the comparison distance, *x*, was reported as being longer than the reference distance:

$$\Psi (x)=\frac{\delta }{2}+(1-\delta )\;\left[\gamma +(1-\gamma )\frac{1}{\sigma \sqrt{2\pi }}\int_{-\infty }^{x}e^{-{\left(t-\mu \right)}^{2}/2\sigma^{2}}dt\right]$$

This function has four free parameters: the mean (*μ*) and standard deviation (*σ*) of the cumulative normal curve, a lapse rate (*δ*), and a y-intercept (*γ*). We allowed *γ* to take on non-zero values, because the psychometric function for many participants did not fall completely to zero at the left tail. Using Bayesian parameter estimation, beginning with uniform prior probabilities over the four parameters, we calculated the joint (*μ, σ, γ*, and *δ*) posterior density. We marginalized this over *δ* and read out the mode of the (*μ, σ, γ*) posterior as the best-estimate of the participant’s psychometric function. We then extracted the comparison distance at which the psychometric function crossed 50% as the perceptual equivalent of the reference distance, i.e., the point of subjective equality (PSE).

## Experiment 2

In Experiment 2, we assessed the extent to which vibratory adaptation changed tactile sensitivity, by measuring participants’ 2IFC detection of force-calibrated Semmes-Weinstein monofilaments (a.k.a von Frey hairs; Timely Neuropathy Testing, LLC and Texas Medical Design, Inc., Dallas, TX, USA). We individually measured the application force produced by each filament with an analytical balance (model AB54-S/FACT, Mettler Toledo).

### Vibrotactile Adaptation Procedure

The participant was seated in front of a table with the experimental apparatus concealed by an opaque black curtain. The participant’s right forearm rested comfortably in a supine position on a padded surface; the wrist was secured to a concave foam support. To mark the skin site for receiving vibratory adaptation, a circle of 19 mm diameter (the size of the adapting probe surface) was drawn with a fine-tipped pen on the volar forearm midway between the wrist and the elbow, and slightly lateral to the proximal-distal midline; the center of the circle was at approximately the midpoint between the two reference points in Experiment 1.

The adapting vibration was delivered via the plastic hemispherical surface of a JVP dome (Stoelting Co., Wood Dale, IL, USA; 19 mm diameter, 0.35 mm groove width). A mechanical arm holding the JVP dome was vibrated via the rotation of an attached eccentric motor (a NexxTech 1.98A DC motor whose axle we asymmetrically weighted, powered at 7.5V by DC power supply 1621A, BK Precision). A force sensor (Honeywell FSG15N1A) in contact with the end of the JVP dome shaft passed a voltage signal proportional to the contact force to an iMac computer via a USB board (NI USB-6210, 16-bit, National Instruments). A custom LabVIEW program monitored the force trace at 5000 samples/s. The program displayed the baseline indentation force and recorded the force waveform during vibration.

To apply the adapting stimulus, the experimenter lowered the mechanical arm and pressed the JVP dome against the participant’s volar forearm at a perpendicular angle. Prior to and during the vibration, the experimenter adjusted the baseline indentation force to approximately 250 g. Post-experiment analysis on the force sensor data showed that the probe vibrated at 122 ± 5 Hz with a peak-to-peak force fluctuation of 125 ± 34 g (mean ± 1 SD; baseline force 245 ± 14 g). As soon as the adapting vibration ceased, the experimenter retracted the mechanical arm to remove the probe from the forearm. The experimenter then applied the monofilament test stimuli. The time between the offset of the adapting vibration and the application of the test stimuli was ~3 s.

### Experiment 2A

To assess the strength of adaptation as a function of vibration duration, we measured participants’ ability to detect Semmes-Weinstein monofilament stimuli applied at the center of the adapted skin site in different adaptation conditions: (a) no-adaptation (NA); (b) 40 s initial adaptation without top-ups (A0); (c) 40 s initial adaptation plus a 3 s top-up vibration prior to each subsequent trial (A3); and (d) 40 s initial adaptation plus a 7 s top-up vibration prior to each subsequent trial (A7). The purpose of the top-ups was to prevent the adaptation effect from waning.

After 20 practice trials with auditory feedback, participants completed the four testing blocks without feedback. Half of the participants completed the four blocks in the order NA-A0-A3-A7, and the other half in the order NA-A7-A3-A0. In the NA-A0-A3-A7 situation, participants took a 10 min break after completing NA, a 10 min break after completing A0, and a 15–20 min break after completing A3. In the NA-A7-A3-A0 situation, participants took a 10 min break after completing NA, a 15–20 min break after completing A7, and a 15–20 min break after completing A3. The breaks after A3 and A7 were longer than after NA or A0, because the A3 and A7 blocks lasted much longer due to the top-ups. The longer breaks were designed to allow participants to recuperate and their nervous systems to recover from possible long-lasting effects of adaptation.

Each testing block had 100 2IFC trials. Each trial consisted of two intervals, separated by ~1.25 s and demarcated by beeps. Simultaneously with one of the beeps, the skin was stimulated with a monofilament for ~0.5 s. By pressing one of two response keys with the left hand, the participant reported whether the stimulus occurred with the first or second beep. Monofilament force began at 0.07 g and was adaptively adjusted via a 2-down 1-up staircase procedure: If the participant answered correctly for two consecutive trials, the monofilament with the next-lower force was applied; if the participant answered incorrectly on any trial, the monofilament with the next-higher force was applied. This procedure converges towards the participant’s 71% correct detection threshold (Levitt, 1971).

At the beginning of each adaptation block (A0, A3 and A7), the circled skin site received a 40 s vibration. Additionally, in the adaptation blocks with top-ups (A3 and A7), the circled site received a 40 s vibration when the participant returned from a break. Within each block, participants took a break after trials 33 and 66. For blocks NA and A0, which occurred relatively quickly, the break duration was 10 s. For blocks A3 and A7, which took much longer because of the top-ups, the break duration was 5 min to allow participants to recuperate.

For each testing block, the participant’s 71% threshold was estimated by averaging the staircase reversal points in the last 50 of the 100 trials. In the rare circumstances in which the last 50 trials contained no reversal points and the participant consistently gave correct responses, so the staircase dropped to and continued at the lowest filament force, we used that force (0.008 g) as the estimated threshold.

### Experiment 2B

To assess the spatial spread of vibrotactile adaptation, we used 40 s adaptation plus 3 s top-ups (protocol A3) and measured 2IFC monofilament detection at four distances from the center of adaptation. In addition to the circle drawn on the participant’s right volar forearm to indicate the site for vibrotactile adaptation, four dots were drawn at 0, 10, 15 and 20 mm from the center of the circle to mark the monofilament test sites. The dots were aligned along the proximal-distal axis of the forearm (Figure 1B). For half of the participants, the dots extended proximally, from the center of the circle towards the elbow; for the other half of the participants, the dots extended distally, from the center of the circle towards the wrist.

Using interleaved 2-down 1-up staircases, we tested the four sites in consecutive trials in the order 0, 10, 15 and 20 mm from the center of the circle. For example, the 0 mm site was tested on trial 1, the 10 mm site on trial 2, the 15 mm site on trial 3, the 20 mm site on trial 4, and the 0 mm site again on trial 5. For all sites, the first trial used the 0.07 g monofilament. The force of the monofilament applied at each test site on subsequent trials followed the staircase procedure based on the participant’s responses at that site. For example, if the participant responded correctly on trials 1 and 5 on which the 0 mm site was tested, then the monofilament applied on the next trial at that site (trial 9) went down to the next-lower force.

After 20 practice trials with auditory feedback, each participant completed two testing blocks without feedback: a NA block and an adaptation (A3) block. Half of the participants completed the NA block first; the other half completed the A3 block first. Each block consisted of 200 trials (i.e., 50 trials at each of the four test sites). In the A3 block, prior to the first trial and every time the participant returned from a break, the circled skin site received a 40 s vibration. To prevent the adaptation effect from waning, the circled skin site received a 3 s top-up vibration prior to each of the subsequent trials. Participants took a 20 min break between testing blocks; within each block, they took a break after completing trials 33, 66, 100, 133 and 166 (break durations: NA block, 10 s after trials 33, 66, 133, 166, 5 min after trial 100; A3 block, 5 min after trials 33, 66, 133, 166, 10 min after trial 100).

For each testing block, the participant’s 71% threshold at each test site was estimated by averaging the staircase reversal points in the last 25 of 50 trials at that site. In the rare circumstances in which the last 25 trials contained no reversal points and the participant consistently gave correct responses, so the staircase dropped to and continued at the lowest filament force, we used that force (0.008 g) as the estimated threshold.

## Experiment 3

In Experiment 3, we investigated the effects of vibratory adaptation on two-point distance perception. We applied the A3 vibrotactile adaptation protocol to the same 20 participants tested in Experiment 2B but on a different day. The participants compared two-point distances on the two forearms, as in Experiment 1, but with or without vibratory adaptation to the intervening skin between the reference points (Figure 1C).

The test skin sites, exclusion criteria, and PSE estimation procedure were as described in Experiment 1. After practice, participants completed three testing blocks, a pre-adaptation (Pre) block without adapting vibration, an adaptation (A3) block, and a post-adaptation (Post) block without adapting vibration. The Pre and Post blocks were identical to the baseline testing blocks in Experiment 1. Participants took a 5 min break after the practice block and a 20 min break between testing blocks. During the Pre and Post blocks, participants took a 1-min break—and during the A3 block, a 5-min break—upon completing each quarter of the 90 trials (i.e., after completing trials 22, 45, 67).

In the A3 block, prior to the first trial and every time the participant returned from a 5 min break, the skin midway between the two reference points (30 mm apart) on the right forearm received a 40 s adapting vibration. In addition, the same skin site received a 3 s vibration as a top-up adaptation prior to each subsequent trial, to prevent the adaptation effects from waning. The adapting probe was removed immediately from the skin when the adapting vibration ceased, and then the two pairs of test stimuli were applied to the forearms successively. The application order of the reference and comparison points was counterbalanced across participants: half of the participants received the reference points first in every trial, and the other half received the comparison points first in every trial. The time between the offset of the adapting vibration and the application of the reference points was ~3 s for participants who received the reference points first, and ~4 s for participants who received the comparison points first.

### Statistical Analyses

We performed ANOVAs with type III sum of squares (and Greenhouse-Geisser correction to the degrees of freedom and the *p*-values in case of violation of sphericity) and two-tailed *t*-tests using SPSS Statistics version 20 (IBM) for Macintosh with an alpha level of 0.05. We performed two-tailed binomial proportion tests in R version 3.0.3. We used R version 3.0.3, companion to applied regression (car) package for *post hoc* one-way repeated-measures ANOVAs. For multiple *post hoc* pairwise comparisons, we used Bonferroni correction and reported *p*-values multiplied by the number of comparisons.

## Results

We undertook a series of three experiments to test for the presence of a tactile adaptation-induced repulsion illusion on the forearm. In a 2IFC task, participants compared the distances of two pairs of point-stimuli (reference vs. comparison) applied on their forearms successively, reporting which distance felt greater. The reference distance was fixed at 30 mm, and the comparison distance varied from 6 mm to 54 mm. The order of the reference and comparison distances was counterbalanced across participants. The PSE (i.e., the comparison distance reported as being greater than the reference distance 50% of the time) was extracted as a measure of participants’ perceived distance between the reference points. We measured baseline PSEs (Experiment 1) and PSEs following vibrotactile adaptation (Experiment 3). We used force-calibrated Semmes-Weinstein monofilaments to assess the efficacy of the adaptation protocol in reducing tactile sensitivity (Experiment 2).

### Baseline Distance Perception Was Unbiased and Stable

In Experiment 1, we assessed the accuracy and stability of participants’ baseline two-point distance perception. Experiment 1 consisted of three identical testing blocks of the 2IFC distance-comparison test without adaptation.

**Figure 3:**
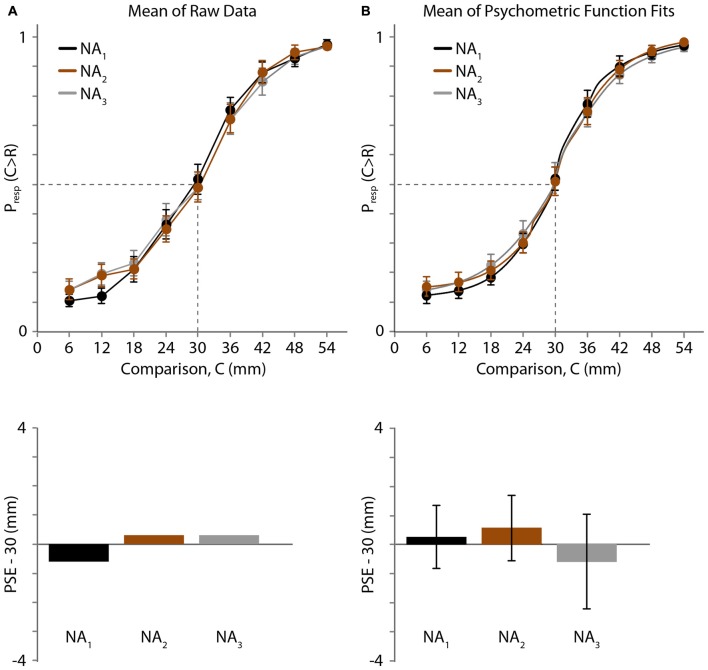
Experiment 1 results. **(A)** Top: mean of raw data (*N* = 19 participants) for three NA blocks. Black, 1st block; brown, 2nd block; gray, 3rd block. Horizontal axis: comparison distance (mm). Vertical axis: proportion of trials in which the comparison distance (C) was perceived as greater than the reference distance (*R* = 30 mm). Dashed lines: P_resp_(C > R) = 0.5 and C = 30 mm. Error bars: ±1 SE (when error bars are not visible, it is because they are smaller than the data point circles). Bottom: for each testing block, the difference between 30 mm and the mean PSE, estimated by linear interpolation of the mean data (top). **(B)** Top: mean of the participants’ individual best-fitting psychometric functions. Error bars: ±1 SE. Bottom: difference between 30 mm and the mean of the PSEs extracted from the participants’ individual best-fitting psychometric functions. Error bars: ±1 SE.

One participant reported that all comparison distances (6–54 mm) were greater than the reference distance (30 mm) in the third testing block; consequently, we could not reliably measure his psychometric curve or PSE for that block. We therefore excluded his data from all three blocks and analyzed the remaining 19 participants’ data. The average psychometric curves and estimated PSEs are shown in Figure 3 (Figure 3A: raw data. Figure 3B: psychometric function fits).

The raw psychometric curves for some participants were noisy and crossed the *y* = 0.5 line multiple times, making it difficult to extract individual PSEs directly from the raw data. Therefore, using the raw data we estimated only the across-participant mean PSE by linearly interpolating the mean response proportions (Figure 3A, top). The mean PSEs obtained in this fashion for the three baseline NA blocks were 29.38, 30.27 and 30.27 mm (Figure 3A, bottom). Binomial tests revealed that the proportion of trials in which participants judged the 30 mm comparison distance as longer than the 30 mm reference distance did not differ significantly from 0.5 for any block (*p* = 0.717, 0.828 and 0.828, for blocks 1, 2 and 3, respectively).

Next, we used Bayesian curve fitting to estimate the psychometric functions and extract the PSEs of the individual participants. Each of the curves shown in Figure 3B (top) is an average of 19 individual best-fitting psychometric curves; the similarity of these three curves to those shown in Figure 3A (top) suggests that our curve fitting procedure provided a valid estimate of participant performance. The means (±1 SE) of the PSEs extracted from the participants’ individual best-fitting psychometric functions for the three blocks were 30.25 ± 1.08, 30.56 ± 1.13 and 29.39 ± 1.62 mm (Figure 3B, bottom). One-sample *t*-tests indicated that none of the PSEs differed significantly from the reference distance of 30 mm (block 1: *t*_(18)_ = 0.227, *p* = 0.823; block 2: *t*_(18)_ = 0.493, *p* = 0.628; block 3: *t*_(18)_ = −0.373, *p* = 0.713), and a one-way repeated-measures ANOVA indicated that the PSEs did not differ across blocks (*F*_(1.485,26.727)_ = 0.458, *p* = 0.580). These results indicate that baseline two-point distance perception was unbiased and stable across testing blocks.

### Focal Vibration Caused a Reduction in Tactile Sensitivity

Having found that participants’ baseline two-point distance comparison judgments were reliable, we next asked whether we could induce focal adaptation between the two reference points. In Experiment 2, we applied prolonged vibration locally to the skin on the reference arm, and we measured 2IFC monofilament detection thresholds as a function of vibration duration and distance from vibration center.

**Figure 4:**
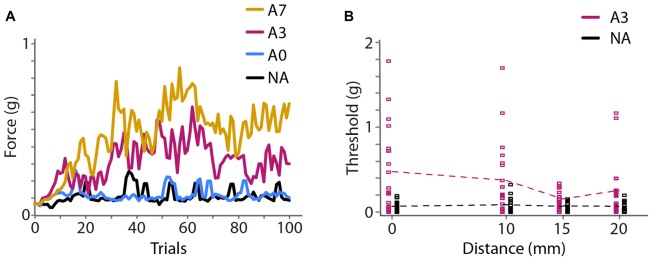
Experiment 2 results. **(A)** Experiment 2A. Mean (*N* = 20 participants) monofilament force applied during 100 trials of a 2IFC detection task following a 2-down 1-up staircase procedure that converges to 71% correct detection. Colors represent different adaptation conditions. Black, NA; blue, 40 s adaptation with no top-ups (A0); magenta, 40 s adaptation with 3 s top-ups (A3); yellow, 40 s adaptation with 7 s top-ups (A7). **(B)** Experiment 2B (*N* = 20 participants). Seventy-one percent correct detection thresholds at four distances from the center of adaptation. Black, NA; magenta, 40 s adaptation with 3 s top-ups (A3). Squares show thresholds of individual participants. Data points from the NA and A3 conditions are slightly offset horizontally for clarity. Dashed lines: mean thresholds.

In Experiment 2A, we found that vibration caused an elevation of monofilament detection thresholds (i.e., a reduction in tactile sensitivity) that increased with the duration of vibration. 71% correct detection thresholds (mean ± 1 SE) at the center of the vibration site were 0.16 ± 0.05 g, 0.20 ± 0.07 g, 0.52 ± 0.12 g and 0.80 ± 0.17 g for the NA, 40 s adaptation, 40 s adaptation with 3 s top-ups, and 40 s adaptation with 7 s top-ups conditions (Figure 4A). A one-way repeated-measures ANOVA indicated a highly significant effect of adaptation duration (*F*_(1.860,35.345)_ = 9.894, *p* < 0.001, partial *η*^2^ = 0.342). *Post hoc* paired-sample *t*-tests comparing each condition to the others revealed that 40 s adaptation alone did not cause significantly different thresholds from the NA baseline condition (*p* = 1.000); however, the addition of a top-up vibration prior to each trial significantly increased detection thresholds. Detection thresholds in the adaptation conditions with 3 s and 7 s top-ups both differed significantly from the NA baseline threshold (3 s top-ups, *p* = 0.015, Cohen’s *d* = 0.676; 7 s top-ups, *p* = 0.006, Cohen’s *d* = 1.182) but did not differ significantly from each other (*p* = 0.608). Thus, 40 s adaptation with 3 s top-ups was sufficient to reduce tactile sensitivity considerably, and the efficacy of this adaptation protocol was comparable to that of a protocol with much longer top-up duration. We therefore chose 40 s adaptation with 3 s top-ups as the protocol to employ in Experiments 2B and 3.

In Experiment 2B, using 40 s adaptation with 3 s top-ups, we found that the threshold elevation was greatest under the adapting probe and diminished as a function of distance (Figure 4B). Seventy-one percent correct detection thresholds (mean ± 1 SE) at the test sites 0, 10, 15 and 20 mm from the center of adaptation were 0.07 ± 0.01 g, 0.08 ± 0.02 g, 0.06 ± 0.01 g and 0.06 ± 0.01 g for the baseline condition, and 0.47 ± 0.11 g, 0.38 ± 0.10 g, 0.15 ± 0.02 g and 0.26 ± 0.07 g for the adaptation condition. A 2 × 4 repeated-measures ANOVA with condition (baseline, adaptation) and distance (0, 10, 15, 20 mm from center of adaptation) as factors indicated a highly significant effect of condition (*F*_(1,19)_ = 24.552, *p* < 0.001, partial *η*^2^ = 0.564), a significant effect of distance (*F*_(3,57)_ = 3.316, *p* = 0.026, partial *η*^2^ = 0.149), and a significant condition × distance interaction (*F*_(2.186,41.542)_ = 3.341, *p* = 0.041, partial *η*^2^ = 0.150). *Post hoc* one-way repeated-measures ANOVAs indicated that the baseline (NA) detection thresholds did not differ across the four distances (*F*_(3,57)_ = 0.854, *p* = 0.470), whereas the detection thresholds in the adaptation condition differed significantly at different distances (*F*_(3,57)_ = 3.381, *p* = 0.024, partial *η*^2^ = 0.151). These results indicate that baseline tactile sensitivity was stable across the forearm test area and that vibratory adaptation effectively reduced tactile sensitivity in a manner that diminished with distance from the center of vibration.

### Focal Adaptation Caused an Illusory Increase in Two-Point Distance

Having established that the adaptation protocol significantly reduced focal tactile sensitivity, we next investigated the effect of focal adaptation on two-point distance perception. In Experiment 3, we measured perceived distance with or without vibrotactile adaptation of the intervening skin between the two reference points.

**Figure 5:**
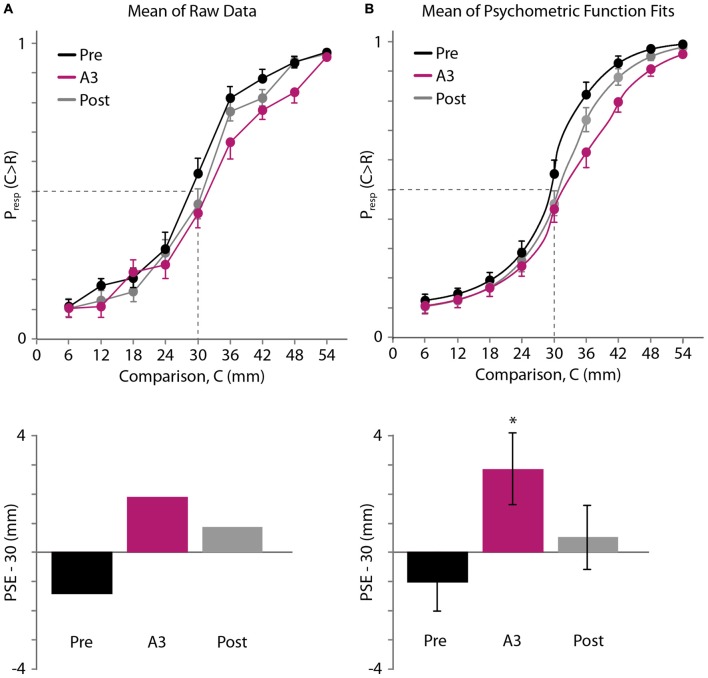
Experiment 3 results. **(A)** Top: mean of raw data (*N* = 20 participants). Black, pre-adaptation (Pre); magenta, adaptation (A3); gray, post-adaptation (Post). Horizontal axis: comparison distance, C. Vertical axis: proportion of trials in which C was perceived to be greater than *R* = 30 mm. Dashed lines: P_resp_(C > R) = 0.5 and C = 30 mm. For visual clarity, error bars show +1 SE, −1 SE and ±1 SE for the highest, lowest, and middle points at each comparison distance, respectively. Bottom: for each testing block, the difference between 30 mm and the mean PSE, estimated by linear interpolation of the mean data (top). **(B)** Top: mean of the participants’ individual best-fitting psychometric functions. Error bars displayed as in **(A)**. Bottom: difference between 30 mm and the mean of the PSEs extracted from the participants’ individual best-fitting psychometric functions. Error bars: ±1 SE. ^*^Significant difference (*p* < 0.05) from 0 mm.

The average psychometric curves and estimated PSEs are shown in Figure 5 (Figure 5A: raw data. Figure 5B: psychometric function fits). As in Experiment 1, we first linearly interpolated the across-participant average of the raw psychometric curves. The mean PSEs obtained in this fashion for the pre-adaptation (Pre), adaptation (A3), and post-adaptation (Post) blocks were 28.59, 31.88 and 30.86 mm, respectively (Figure 5A, bottom). Binomial tests revealed that the proportion of trials in which participants judged the 30 mm comparison distance as longer than the 30 mm reference distance did not differ significantly from 0.5 for the Pre (*p* = 0.104) and Post blocks (*p* = 0.229). In contrast, this proportion did differ from 0.5 for the A3 block (mean proportion, 0.425; *p* = 0.040). These results are consistent with a rightward shift of the psychometric curve.

Next, we used Bayesian curve fitting to estimate the psychometric functions and extract the PSEs of the individual participants. Each of the curves shown in Figure 5B (top) is an average of 20 individual best-fitting psychometric curves; the similarity of these three curves to those shown in Figure 5A (top) suggests that our curve fitting procedure provided a valid estimate of participant performance. The means (±1 SE) of the PSEs extracted from the participants’ individual best-fitting psychometric functions for the Pre, A3, and Post blocks were 28.98 ± 1.00, 32.85 ± 1.23, and 30.51 ± 1.10 mm (Figure 5B, bottom). One-sample *t*-tests indicated that the A3 PSE was significantly greater than the reference distance of 30 mm (*t*_(19)_ = 2.322, *p* = 0.031, Cohen’s *d* = 0.519). By contrast, neither the Pre PSE nor the Post PSE differed significantly from 30 mm (Pre PSE: *t*_(19)_ = −1.024, *p* = 0.319; Post PSE: *t*_(19)_ = 0.461, *p* = 0.650). A one-way repeated-measures ANOVA indicated that the PSEs differed significantly across conditions (*F*_(1.412,26.835)_ = 5.643, *p* = 0.016, partial *η*^2^ = 0.229). *Post hoc* paired-samples *t*-tests indicated that the A PSE differed from the Pre PSE (*p* = 0.021, Cohen’s *d* = 0.886), whereas the Post PSE did not differ from the Pre PSE (*p* = 0.129) or from the A PSE (*p* = 0.319). These results indicate that focal vibrotactile adaptation increased the perceived distance between two points straddling the adapted skin area.

## Discussion

We have reported an adaptation-induced tactile spatial repulsion illusion: vibrotactile stimulation focally reduced tactile sensitivity and increased the perceived separation between points straddling the adapted region. Whereas adaptation-induced spatial illusions have been well studied in vision, and to a lesser extent in audition, such illusions have rarely been reported in touch. Our finding suggests that adaptation plays a central role in calibrating spatial perception in multiple sensory modalities.

### Comparison to Previous Tactile Adaptation Studies

Previous studies have characterized the effects of tactile adaptation on amplitude detection threshold, intensity estimation, amplitude and frequency discrimination, and motion direction and speed perception (Hahn, 1966, 1968; Gescheider and Wright, 1968; Berglund and Berglund, 1970; Hollins et al., 1990; Goble and Hollins, 1993, 1994; Tommerdahl et al., 2005; Tannan et al., 2007; McIntyre et al., 2016a,b). By contrast, the effects of adaptation on tactile spatial perception have been rarely studied. In one of the few modern studies in this area, Tannan et al. (2006) tested participants’ ability to identify which of two skin locations on the dorsal hand was tapped. Following 5 s of 25 Hz sinusoidal skin displacement to one of the stimulus sites, participants’ accuracy improved. Tannan et al. (2006) interpreted their finding to indicate that the adaptation caused an improvement in spatial acuity, perhaps because it resulted in more clearly defined loci of activation in the primary somatosensory cortex (SI). However, a plausible alternative hypothesis is that a test stimulus applied to the adapted site felt weaker than one applied to the non-adapted site, and that this intensity cue caused the increased accuracy on the task. Very recently, Calzolari et al. (2017) reported that adaptation to specific tactile distances can lead to spatial aftereffects. The authors repeatedly applied 2-point stimuli separated by short distances to one hand and 2-point stimuli separated by long distances to the other hand. Exposure to short-distance stimuli caused subsequent stimuli on that hand to appear longer, and exposure to long-distance stimuli caused subsequent stimuli to appear shorter. This interesting perceptual repulsion phenomenon may be of a different nature than the adaptation-induced repulsion that we have observed, as the adapting stimuli in Calzolari et al. (2017) were themselves two-point stimuli, and the authors intentionally varied the stimulus positions on each hand from trial to trial in order to avoid adapting specific skin locations. In contrast, we applied a vibratory stimulus to the intervening skin region between two points precisely in order to adapt that specific area.

In an early study of adaptation-induced tactile repulsion, Silver (1969) reported a tactile equivalent of the visual tilt aftereffect illusion. In the visual tilt aftereffect illusion, prolonged viewing of oriented bars causes subsequently viewed bars of nearby orientation to appear tilted away from the adapting orientation (Gibson and Radner, 1937; Blakemore, 1973). Silver (1969) reported that 1 min of static indentation or active scanning of a tilted bar resulted in an analogous repulsive aftereffect in tactile orientation perception. This study was reported in a doctoral dissertation; unfortunately, to the best of our knowledge, the study did not appear in any later peer-reviewed report.

Two other early studies (Day and Singer, 1964; Gilbert, 1967) had strong similarities to the present study. In both studies, participants compared the perceived distance between two parallel bars pressed transversely against one forearm (the reference arm) with two similar bars pressed against the other forearm. In the adaptation conditions, static pressure was applied for 90 s before the first trial with a 10 s top-up before each of the subsequent trials. The adapting stimulus was applied either on the intervening skin between the reference bars (“inside adaptation”) or on the adjacent skin outside the reference bars (“outside adaptation”). The perceived distance between the reference bars increased following adaptation of the intervening skin (Day and Singer, 1964) and decreased following adaptation of the adjacent outside skin (Day and Singer, 1964; Gilbert, 1967). Both results indicated that the bars were perceptually shifted away from the adapted skin regions, a repulsion illusion consistent with our findings. However, the proper interpretation of these studies’ results is somewhat unclear. Gilbert (1967) argued that the apparent repulsion effect reported by Day and Singer (1964) owed primarily to the authors’ use of different ranges of comparison distances for the “inside” and “outside” adaptation conditions, ranges that were not symmetrically distributed about the reference separation; the exposure to particular distributions of comparison distances may have resulted in a recalibration of the perception of distance, a sort of statistical adaptation described previously by Helson (1947). Gilbert (1967) suggested that, when this factor was taken into account, little evidence remained for a true repulsion effect in either study.

In light of this controversy, we revisited the question of whether adaptation-induced spatial repulsion occurs on the forearm. We used comparison separations that were symmetrically distributed around the reference separation and found clear evidence for tactile repulsion similar to the “inside adaptation” repulsion effect reported by Day and Singer (1964). The adapting and test parameters used in our study differed from those used by Day and Singer (1964) and Gilbert (1967). Specifically, in our study, the adapting stimulus was a vibration rather than static pressure; the duration of the adapting stimulus was shorter; our test stimuli were much smaller in size (1.5 mm diameter spherical points instead of 30 × 1.5 mm bars); and our test stimuli were much closer together (30 mm instead of 110 mm). The similar perceptual effects observed in our study and these two early studies suggest that adaptation-induced tactile spatial repulsion is robust to variability in adapting and test parameters.

Additional research is needed to determine the duration of the adaptation-induced repulsion effect. A curious aspect of our Experiment 3 is that the post-adaptation psychometric function appeared not to fully recover to the baseline state. This result was not statistically significant, as the post-adaptation PSE did not differ significantly from 30 mm (Figures 5A,B). Nevertheless, the possibility exists that our participants experienced some residual adaptation effect 20 min after the adaptation phase ended. To the best of our knowledge, no psychophysical or neurophysiological studies have reported such a long recovery time following merely tens of seconds of vibrotactile adaptation and seconds of top-ups. For instance, Hahn (1966) reported that, after 25 min of continuous vibrotactile adaptation at 200 μm peak-to-peak amplitude and 60 Hz, recovery largely occurred (as measured by psychophysical threshold or amplitude matching) within the first 1–2 min and fully completed after 8–12 min. A possible explanation for the discrepancy is that we used an intense adapting stimulus (125 g peak-to-peak force) that likely adapted multiple types of tactile channels (Bensmaia et al., 2005; Leung et al., 2005). Future studies are needed to characterize the time course of the adaptation-induced spatial repulsion effect and how it is affected by characteristics of the adapting stimulus.

### At What Level(s) of the Somatosensory System does Focal Adaptation Act to Cause the Repulsion Illusion?

Where in the somatosensory processing pathway does the adaptation take place that leads to the perceptual repulsion observed in the present study? A difficulty in discerning the relevant neural locus of adaptation is that neuronal responses will reflect changes in the driving input from earlier processing levels. Indeed, a general conclusion from the visual literature is that adaptation can exert effects—either direct or indirect—at multiple processing stages (Kohn and Movshon, 2003, 2004; Kohn, 2007; Dhruv and Carandini, 2014). For instance, under a variety of stimulus scenarios, adaptation results in changes in both subcortical and cortical neural responses. Similarly, in the tactile system, exposure to sustained vibration leads to lasting reduction in neural responsivity in the PNS (Bensmaia et al., 2005; Leung et al., 2005) and CNS (Bystrzycka et al., 1977; O’Mara et al., 1988; Whitsel et al., 2001).

A few somatosensory studies have provided convincing evidence for a strong central contribution to adaptation by comparing the degree of adaptation that occurs at multiple levels of the processing hierarchy. O’Mara et al. (1988) recorded extracellular responses of PC afferents and cuneate neurons to 300 Hz sustained vibration in decerebrated or anesthetized cats. It was found that: (1) afferent-induced inhibition was too brief to account for the long-lasting response depression in cuneate neurons; and (2) for cuneate neurons that received excitatory input from multiple skin sites, following 300 Hz adapting vibration on one site, the neurons displayed lasting response depression to 30 Hz test vibration on an unadapted site. O’Mara et al. (1988) concluded that peripheral factors make little contribution to the lasting adaptation effects observed in central neurons, and therefore presumably little contribution to adaptation effects at a perceptual level. Support for a central locus of vibrotactile adaptation was similarly provided by Whitsel et al. (2003). These investigators recorded responses of rapidly adapting (RA) afferents and SI RA neurons to sustained 10–50 Hz flutter stimulation in anesthetized monkeys and cats. Under the same stimulus condition, RA cortical neuron responses declined to a much greater extent than RA afferent responses. Finally, Chung et al. (2002) recorded simultaneously from neurons in the rat somatosensory thalamus and barrel cortex in response to repetitive brief whisker deflection, and found that the cortical responses declined more strongly, more quickly, and recovered more slowly than the thalamic responses. Chung et al. (2002) concluded that both subcortical and cortical mechanisms contributed to adaptation, and they suggested that rapid depression of thalamocortical synapses played a key role in cortical adaptation.

Intrinsic signal optical imaging studies in SI have shown that 1–10 s of flutter stimulation on the skin increased the absorbance of regions in areas 3b and 1 that received input from the stimulated skin site and decreased the absorbance of surrounding regions (Tommerdahl et al., 2002; Simons et al., 2005, 2007). The altered activities did not return to baseline levels until 10–15 s after stimulus offset. The results indicated that flutter adaptation narrows the spatial extent of SI response to a sustained stimulus; this sharpening has been proposed to underlie the enhancement of spatial discrimination following flutter adaptation (Tommerdahl et al., 2002, 2005). Another intrinsic signal optical imaging study showed that, in response to sustained 200 Hz vibration, SI initially exhibited a transient increase in absorbance that dropped to below-background level after 1 s, whereas the secondary somatosensory cortex (SII) exhibited a vigorous and well-maintained increase in absorbance (Tommerdahl et al., 1999). Although the perceptual consequences of such responses of SI and SII to vibrotactile adaptation are unknown, the results suggest that vibrotactile adaptation shapes cortical response dynamics. Last but not least, a functional MRI study in humans showed that the number of activated voxels in SI and SII exponentially reduced over time in response to 15 s of static pressure on the fingertip (Chung et al., 2015). Chung et al. (2015) interpreted the results as suggesting that cortical activation is refined during tactile adaptation. The converging evidence of substantial cortical changes during prolonged tactile stimulation suggests a cortical locus of adaptation; however, it is important to keep in mind the caveat that observed cortical changes could reflect subcortical adaptation.

In contrast to the above studies, a recent, intriguing perceptual study in humans reported that vibrotactile adaptation occurs predominantly at the peripheral level. Klocker et al. (2016) performed experiments with either vibrotactile stimulation or transcutaneous electrical stimulation. They assumed electrical stimulation would bypass peripheral mechanoreceptor transduction and activate primary afferent axons directly. Klocker et al. (2016) reasoned that, if vibrotactile adaptation induced changes at a central level, then prolonged mechanical vibration on the fingertip would affect the ability to detect not only vibration but also electrical impulses on the fingertip. Contrary to this prediction, they found that vibrotactile adaptation of the fingertip impaired only vibration detection, leaving electrical detection intact. Similarly, prolonged electrical stimulation of the median nerve—which should have induced central adaptation—did not affect subsequent vibration detection on the fingertip. Klocker et al. (2016) concluded that somatosensory adaptation occurs predominantly in peripheral mechanoreceptors. Unfortunately, the authors did not report whether electrical adaptation of the median nerve impaired electrical detection on the fingertip. A plausible alternative hypothesis is that the vibratory and electrical stimuli activated different cutaneous channels, and adaptation of one channel did not affect perception via the other.

If peripheral adaptation contributes to the illusion we have reported, a second question of interest is: which mechanoreceptive afferents are involved? Five mechanoreceptive channels that convey action potentials via fast-conducting Aβ fibers have been identified in human forearm skin: slowly-adapting type 1 afferents (SA1), slowly-adapting type 2 afferents (SA2), and three fast-adapting types: hair units, field units, and Pacinian (PC) units (Vallbo et al., 1995; Olausson et al., 2000). Our intense adapting vibration (peak-to-peak amplitude ~125 g, frequency ~122 Hz) and strong test stimuli (point static pressure, ~80 g) likely activated multiple types of afferents (Bolanowski et al., 1988; Abraira and Ginty, 2013) and caused adaptation in them as well (Bensmaia et al., 2005; Leung et al., 2005). Evidence suggests that, as in glabrous skin (Johnson, 2001; Abraira and Ginty, 2013), in forearm skin only SA1s have the characteristics that are needed to convey fine spatial information. SA1s innervate the human forearm close to the skin surface and are highly responsive to light skin indentation; they have small, distinctive receptive fields and high distribution density compared to the forearm’s fast-adapting afferents (Vallbo et al., 1995; Olausson et al., 2000). These characteristics suggest that the spatial pattern of SA1 firing rates encodes the spatial structure of stimuli pressed against the skin. Like SA1s, SA2 afferents are sensitive to local skin strain, and some microneurography studies have estimated that in human forearm skin the size and distribution density of SA2 receptive fields are comparable to those of SA1s (Vallbo et al., 1995; Olausson et al., 2000). However, SA2s are characterized by continuous spontaneous firing, pronounced enlargement in receptive field size with stronger stimulation, and high sensitivity to directional horizontal skin stretch (Chambers et al., 1972; Edin, 1992; Vallbo et al., 1995; Olausson et al., 2000). Interestingly, intraneural activation of individual SA2 afferents, unlike stimulation of other afferent types, did not evoke conscious sensation (Ochoa and Torebjörk, 1983). Given their response characteristics, SA2s are presumably better suited for proprioceptive signaling than for conveying fine spatial information. The fast-adapting afferent types (hair units, field units, and PCs) presumably do not contribute significantly to fine spatial coding, as they have large receptive fields with diffuse borders and low distribution density (Bolanowski et al., 1994; Vallbo et al., 1995). As SA1s have the requisite properties to serve fine spatial perception on the forearm, it is likely that the perception of two-point distance relies largely on SA1 input, and it is plausible that adaptation in the SA1 population contributes to the repulsion illusion reported here.

### A Model for Tactile Spatial Localization and Adaptation-Induced Aftereffects

**Figure 6:**
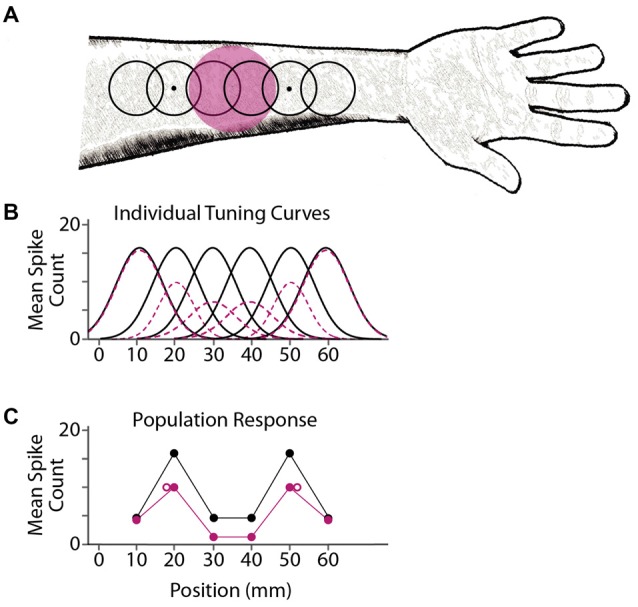
A model for adaptation-induced tactile spatial aftereffects. **(A)** Black open circles depict receptive fields of six simulated cortical neurons, with centers at 10, 20, 30, 40, 50 and 60 mm on the arm. Dots depict two point-stimuli delivered simultaneously at 20 and 50 mm. Magenta circle depicts the area adapted by prolonged tactile stimulation (e.g., vibration). **(B)** Mean firing (spike counts) expected from the six individual neurons in response to a point stimulus at each location on the *x*-axis, before adaptation (black solid curves) or following adaptation (magenta dashed curves). For illustration purposes, these simulated tuning curves are Gaussian functions; the actual shapes, sizes, and activity profiles of cortical receptive fields are much more variable than shown here (Peters et al., 2015). **(C)** Simulated population response from the six neurons. Each solid circle depicts a neuron’s mean firing rate (vertical axis) plotted against the neuron’s receptive field center location (horizontal axis). Black, NA; magenta, adaptation. The two point-stimuli evoke two mounds of activity in the population response. Open magenta circles: focal adaptation shifts the perceived locations of the stimuli (e.g., the average of the receptive field center locations in each mound, weighted by the firing rates of the neurons) away from the adapted area. The brain consequently misattributes the focal reduction in firing to a greater distance between stimulus points.

We propose that tactile stimulus localization is based on responses from a population of neurons with overlapping receptive fields. Prolonged exposure to a focal stimulus reduces the responsiveness of nearby neurons, via either fatigue (Köhler and Wallach, 1944; Sutherland, 1961; Barlow and Hill, 1963) or lingering inhibition (Ganz, 1966; Tolhurst and Thompson, 1975; Magnussen and Kurtenbach, 1980), resulting in a shift in perceived location (Figure 6). Inherent in this model is the hypothesis that the brain interprets stimulus-evoked neural activity without accounting for the fact that the neurons are in a state of adaptation.

From an information processing perspective, perception in touch as in other modalities can be viewed as consisting of two fundamental stages: encoding and decoding (Pouget et al., 2000; Paninski et al., 2007; Peters et al., 2015). The encoding stage samples sensory stimuli from the environment and converts these into spatiotemporal patterns of action potentials. This forward processing or data generative stage is stochastic, both because natural sensory stimuli are samples from an environmental stimulus distribution and because individual neurons respond stochastically (e.g., with Poisson variability; Sripati et al., 2006). The decoding stage interprets the observed action potential pattern in an attempt to infer the stimulus that caused it. The decoder is thus undertaking the notoriously difficult inverse problem of inferring a cause from its stochastically generated effects (Pizlo, 2001). As a consequence, perceptual inference is inherently uncertain.

An ideal Bayesian observer would decode by interpreting the observed action potential pattern in light of the actual generative model; that is, optimal perceptual inference requires that the observer’s prior probability distribution match the actual stimulus distribution and that the observer’s likelihood function correctly represent the statistical mapping from stimuli to observation (Ma, 2012). We propose that violation of the second of these conditions underlies adaptation-induced repulsion illusions such as the one reported in the present study. Specifically, in keeping with similar suggestions from the visual and multisensory literature, we propose that focal adaptation leads the brain to mistakenly infer that a subsequently presented stimulus is shifted away from the adapted region, because the brain decodes without taking into account that the sensory system is focally adapted (Schwartz et al., 2007; Series et al., 2009; Crommett et al., 2017; Figure 6). Future research will apply Bayesian modeling to further investigate the tactile repulsion illusion reported here.

## Conclusion

The current study supports the similarity of spatial processing in touch, vision and audition. Previous studies have revealed similarities between tactile and visual processing for perception of spatial properties such as orientation, shape and form (Phillips et al., 1983; Hsiao, 1998; Bensmaia et al., 2008; Yau et al., 2009). The study of spatial illusions has also revealed similar processing across sensory modalities. Notably, tactile, visual and auditory perception all are prone to perceptual length contraction illusions (e.g., sensory saltation) that occur in response to discrete stimuli delivered in rapid succession (Geldard, 1982; Goldreich, 2007; Getzmann, 2009; Khuu et al., 2011; Goldreich and Tong, 2013; Tong et al., 2016). In the current study, we have verified that another type of spatial illusion, adaptation-induced spatial repulsion, which has been demonstrated previously in vision and audition (Thurlow and Jack, 1973; Kashino and Nishida, 1998; Carlile et al., 2001; Clifford et al., 2007; Kohn, 2007; Schwartz et al., 2007), occurs also in touch. Collectively, these observations suggest that spatial processing operates via fundamentally similar mechanisms in different sensory modalities.

## Author Contributions

LL and DG designed the study. LL, AC and SMI conducted the experiments. AC and SMI contributed to preliminary data analysis and draft reports. LL and DG performed the final data analyses and wrote the article with feedback from AC and SMI.

## Conflict of Interest Statement

The authors declare that the research was conducted in the absence of any commercial or financial relationships that could be construed as a potential conflict of interest.
